# The Perspective of Nectarine Fruit as a Sugar Substituent in Puddings Prepared with Corn and Rice Starch

**DOI:** 10.3390/foods10112563

**Published:** 2021-10-24

**Authors:** Dasha Mihaylova, Aneta Popova, Zhivka Goranova, Dorina Petkova, Pavlina Doykina, Anna Lante

**Affiliations:** 1 Department of Biotechnology, Technological Faculty, University of Food Technologies, 4002 Plovdiv, Bulgaria; dashamihaylova@yahoo.com (D.M.); dorina_petkova@abv.bg (D.P.); 2 Department of Catering and Nutrition, Economics Faculty, University of Food Technologies, 4002 Plovdiv, Bulgaria; pavlina_doikina@abv.bg; 3 Institute of Food Preservation and Quality, 4002 Plovdiv, Bulgaria; jivka_goranova@abv.bg; 4 Department of Agronomy, Food, Natural Resources, Animals, and Environment—DAFNAE, Agripolis, University of Padova, 35020 Legnaro, Italy; anna.lante@unipd.it

**Keywords:** puddings, starch, nectarine, novel ingredient, sensory properties, rheology

## Abstract

It has been long recognized that fruits are healthy diet compounds as they are excellent sources of health-beneficial bioactive components (polyphenols, minerals, vitamins, organic acids, etc.). The diversification of the consumer’s taste calls for an expansion of food options and novel ingredients. Puddings are a well-known food choice introduced in the human diet at a very early age because of their easy and high digestion. Four formulations with two types of starch (corn and rice) were selected as object of analysis. Nectarines were incorporated as a purée, and lyophilized powder. The nectarine variety “Gergana”, used for the preparations, is a local variety with proven beneficial properties. The study aimed at analyzing the physical (moisture, ash, color, water-holding capacity, water activity, density and syneresis), textural (firmness, gumminess, cohesiveness, springiness, and chewiness), nutritional, and sensory characteristics of the nectarine-enriched puddings. The outcomes obtained from this study provided significant information about the possible application of the formulations in the children’s daily menus. All four formulations had distinct peachy aroma. The formulations prepared with nectarine purée resulted in a better sensory perception about their texture, and better water-holding capacity. At this point, the formulation prepared with lyophilized fruit and rice starch has the most promising results. Sufficient evidence leads to further exploration of the perspective of fruit-enriched puddings in order to improve their technological and health-promoting properties.

## 1. Introduction

Desserts have always been seen as an accessory to special occasions, and are usually associated with a sweet taste. Throughout history, fruits like figs, dates, and grapes, are traditionally used in desserts. Fruits are generally eaten without adding sugar, and manage to fulfill the feeling of sweetness in the absence of refined sugar.

Functional ingredients in foods are seen as a trending direction of sustainable food production especially linked to the incorporation of natural raw ingredients [1]. The advanced consumer knowledge about foods with potential health benefits results in the development of new products with health-promoting ingredients [2]. Hunger satisfaction is only a small part of the contemporary food production chain. Plant biochemistry and its recent findings advocate for fruits’ naturally health-promoting substances [3]. A strong interest in foods with functional properties for children exists [4].

Milk-based desserts are one of the most popular ones to all age groups. They are simple in preparation and usually comprise of milk (skimmed or whole), thickener (usually starch or hydrocolloid), colorant, aroma, and sugar or sugar substitutes (stevia, agave, honey, etc.). The texture properties of such products are directly linked to their consumer’s acceptance. Alteration of the original recipe calls for research of the rheological properties and sensory attributes of the newly developed product. Depending on the product, texture or flavor is the most important characteristic speaking to the consumer’s needs [5].

All products are created to be able to withstand the consumer’s need as well as the market’s demands. Recently, the food industry has been making efforts toward the development of products with functional properties, as contemporary consumers are seeking to maximize the beneficial effect of the food they daily intake. Research shows that common dessert recipe alterations are milk substitutes, due to the rising cases of protein allergies and enzyme deficiencies [6]; prebiotic polysaccharides enrichment [7]; hydrocolloid alternatives [8]; sugar and fat content reduction [9]. The continuously growing consumption of sugar rich food products leads to the distinctive preference for the sweet flavor. Puddings are a well-known food choice introduced in the human diet at a very early age because of their easy and high digestion. The limited number of ingredients makes them particularly suitable for children. Carbohydrates and starches in particular, are primary dietary sources of energy to support children’s development [10]. When evaluated from the point of its botanical source, starch can be extracted from rice, maize, tapioca, wheat, banana, etc. Rice starch, for example, is hypo-allergenic, because of the non-allergenicity of the associated protein [11]. In the gastrointestinal tract, α-amylase mainly aids in the digestion of starch and its conversion into olygosaccharides [12]. Consequently limit dextrinase, glucoamylase, maltase, sucrase, and lactase act on the further absorption of starch. Efforts have been made to understand the infant’s formula disintegration mechanisms in order to be able to develop appropriate new formulations with health benefits [13].

Cow milk can be seen as food allergen especially considering the child’s reaction to casein and β-lactoglobulin [14]. However, a proper balanced child diet includes milk because of its high protein quality and vitamin availability [15]. Depending on each country’s recommendations, the introduction of dairy products can be made at a different age, but it is usually in the period from 8 to 12 months.

The excessive refined sugar intake has always been seen as one of the major issues leading to the manifestation of obesity, hyperlipidemia, hyperglycemia, diabetes type 2, and cardiovascular issues. The insufficient physical activity and poor nutritional choices is a prerequisite to unhealthy weight gain at an early age [16]. Many EU countries are advising on the reduction of sugar intake in the children’s daily menu as the overweight statistics continue to grow [17]. The increased cases of non-communicable diseases challenges technologists to provide desserts with desired sensory attributes and a reduced calory count. A possible approach is, using ingredients that can mimic a certain taste, but actually provide added value to the dish in terms of health-promoting substances.

The sweet taste is in fact the first recognizable taste by the child due to the taste of breastmilk. The sweet taste is subconsciously linked to safety and happiness [18]. The sweet taste is also linked to energy providing because, from a physiological point of view, the human body breaks food into simple glucose in order to provide energy. Recent research shows that labelled as healthier products (with no sugar, chocolate, etc.) are considered not very tasty and less preferred [19].

The full or partial substitution of sugar in different desserts is not new [20]. In most cases, the substitution is at the expense of honey or other natural or artificial sweeteners. It has been long recognized that fruits are healthy diet compounds as they are excellent sources of health-beneficial bioactive components (polyphenols, minerals, vitamins, organic acids, etc.). Carbohydrates in fruit are usually represented by sucrose, glucose, and fructose [21]. This, combined with their appealing aroma and naturally likeable flavor, makes them a promising accessory to a healthy diet. Fruits can be found as components of desserts both fresh and dried. When a child-oriented dessert is designed all possible allergy borne fruit like berries, citric fruits, peaches with fuzz, pineapples, etc. [22] should be avoided as a possibility.

Modern cuisine is focusing on products that can bring a pleasurable taste at the expense of natural products, with the use of healthier alternatives not only in terms of the technology being used (vacuum cooking, hot infusion, filtration, etc.), but also with the exploitation of natural products.

The aim of the study was to evaluate the incorporation of nectarines as lyophilized powder and purée, in puddings prepared with corn and rice starches in order to meet the needs of the trending healthy lifestyle and diet. The physical (moisture, ash, color, water-holding capacity, water activity, density and syneresis), textural (firmness, gumminess, cohesiveness, springiness, and chewiness), nutritional, and sensory characteristics of the nectarine-enriched puddings were used to describe the newly developed formulations.

## 2. Materials and Methods

### 2.1. Materials

Fresh nectarine samples of the “Gergana” variety were provided from the Fruit Growing Institute, Plovdiv, Bulgaria. Samples from the same batch were also lyophilized. Corn and rice starches used in the current study were purchased from “Balev Bio Market” shop (Plovdiv, Bulgaria). Both starches are produced and packaged (120 g) by CLECA S.p.A., Italy. The whole (3%) cow milk produced and packaged by “Jaltusha” dairy, Ardino village, Kardzhali region, Bulgaria, was purchased at a local store in Plovdiv, Bulgaria.

### 2.2. Preparation of Puddings

The puddings were prepared in laboratory conditions at the University of food technologies. Table 1 provides information about the percentage distribution of the ingredients used to prepare the formulations.

**Figure 1:**
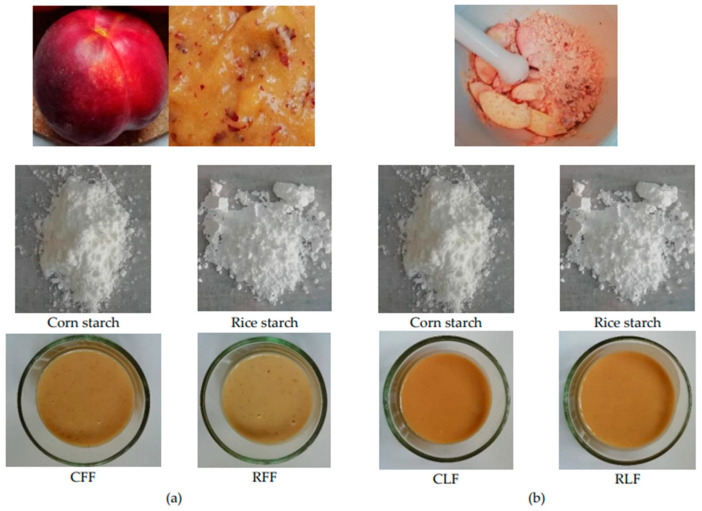
Pudding formulations. (**a**) RFF—rice starch, peach purée; CFF—corn starch, peach purée; (**b**) RLF—rice starch, lyophilized fruit; CLF—corn starch, lyophilized fruit.

The fresh nectarine was puréed using Muhler Nutritional blender MNB-688. The lyophilized fruit was powdered with Tefal GT110838 grinder. The milk and fruit was mixed at appropriate quantities and heated up to 50 °C before the starch powder was added. The resulting mixture was heated to 85 °C with constant stirring and these conditions were retained for 2–3 min. The formulations (Figure 1) were then poured into containers according to their further usage, and cooled down to 25 °C.

### 2.3. Ash Content

Ash content was determined by burning in a muffle furnace according to AOAC 945.46 [23].

### 2.4. Moisture Content

Total moisture content of the samples was determined according to the procedure described in AACC method 44-15A [24].

### 2.5. Nutritional Data

The nutritional data was determined by the calculation method. The nutritional value of the finished products was calculated per 100 g based on specifications obtained from suppliers of each of the ingredients (starch, milk). The nutritional value of the “Gergana” nectarine was based on previous research [25].

### 2.6. Color

The color was analyzed with the use of PCE-CSM 2 (PCE-CSM instruments, Deutschland) with a measuring aperture of 8 mm. The L * (lightness; ranging from 0 to 100), a (red-green coordinate), b (yellow-blue coordinate), chroma (color saturation), and hue angle (color tone) were evaluated.

### 2.7. Water Holding Capacity

Water holding capacity (WHC) was defined as described by Raungrusmee and Anal [26] with modifications. 20 g of each formulation (W_2_) was placed in a 50 mL centrifuge tube, and the tube was centrifuged for 20 min at 5000 rpm. The supernatant (W_1_) was decanted. WHC (%) was calculated following the equation:

WHC = (1 − W_1_/W_2_) × 100 (1)

### 2.8. Freeze-Thaw Stability (Syneresis)

Freeze-thaw stability was evaluated with modifications as described by Wang et al. [27]. Samples were stored at 4 °C for 16 h, frozen at −18 °C for 24 h, and at 25 °C for 4 h. Then the sample formulations were placed in a funnel allowing the water to drop by gravity for 2 h. The amount of water released from the gel was measured by weighing and expressed as percent of water separated (syneresis).

### 2.9. Water Activity

The water activity (a_w_) of the obtained formulations was measured with a Rotronic HP23-AW-A Lachen, Bassersdorf, Switzerland.

### 2.10. Density

Density was measured in a temperature regulated room. A 10 cm^3^ graduated cylinder where the pudding was placed, was used to measure the difference in the volume to its mass, used to express the density parameter. Density was expressed as the ratio of the mass of the sample to its volume in g/cm^3^.

### 2.11. Texture Profile Analysis (TPA)

**Figure 2:**
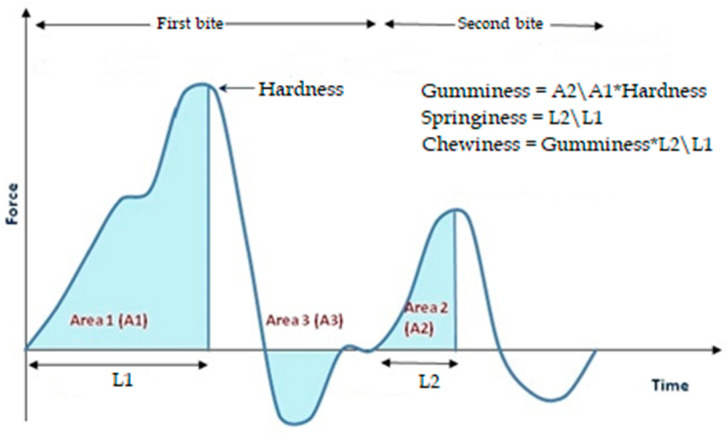
TPA parameters.

TPA was performed by Texture Analyzer (Stable Microsystems, TAXT-2i Texture Analyzer, Godalming, Surrey, UK) with 0.05 N load cell, as described by Vidigal et al. [28]. The texture parameters (firmness, gumminess, chewiness, springiness, and cohesiveness) were determined in a texture profile analysis mode (TPA) with 50 mm diameter cylinder probe, 20% strain of penetration, 5 s of waiting time, and 2 mm/s of test speed, penetration distance was 15 mm (50% of the sample height) (Figure 2).

### 2.12. Sensory Evaluation

Sensory analysis was performed at the University of Food Technologies. The sensory panel of 10 specially selected evaluators was recruited following ISO 8586 [29] based on their eating habits, desserts in particular. All participants in the evaluation have passed the preliminary tests to assess main tastes and are familiar with the sensory test methodology. The four male and six female panelists used a modified Sensory Spectrum method, and objective method for describing the intensity of attributes to references. A lexicon of attributes was developed for the needs of the evaluation. The pudding formulations were evaluated by a 15-point ascending scale.

Each pudding formulation was labelled with a 3-digit code in a randomized design. Panelists cleansed their palates between samples using water and crackers, and evaluated the puddings for appearance (*n* = 5), basic taste (*n* = 3), texture (*n* = 4), and aroma (*n* = 4). The phase of the evaluation followed international standards ISO 6564 [30] and ISO 4121 [31] as well as techniques described by Lawless et al. [32] and Meilgaard et al. [33].

### 2.13. Statistical Analysis

Data were analyzed using MS Excel software. All assays were performed in at least three repetitions. Results were presented as mean ±SD (standard deviation). Relevant statistical analyses of the data were performed using one-way ANOVA and a Tukey–Kramer post hoc test (α = 0.05), as described by Assaad et al. [34]. The Pearson’s correlation coefficients between moisture content, density, a_w_ and textural parameters have been plotted with the use of Excel STAT Cloud function in Microsoft Excel 365 software.

## 3. Results and Discussion

Fruits have long been recognized as healthy attributes to the daily human diet. They contain several beneficial substances such as minerals, vitamins, organic acids, polyphenols, etc. [35]. The genus Prunus comprises of more than 50 species and accepted taxa [36]. The different nectarine varieties are nutritionally important, and can provide the human body with vitamin A, C, E, carotenoids and flavonoid polyphenolic antioxidants [37]. Nectarines continue to gain popularity due to their smooth structure and favorable aroma, and color. The absence of fuzz on the peach’s skin is directly linked to it not provoking an initial allergic reaction.

The relatively low consumption of fruit worldwide calls for their incorporation in the daily diet not only as fresh components but also as ingredients to different products. Fruits are one of the first single food components introduced to infants [38]. The currently developed formulations rely on lyophilized nectarines which are accessible year round, and fresh nectarine purée used during the peach season, from June until October depending on variety availability.

### 3.1. Ash and Moisture Content of the Nectarine-Enriched Puddings

Different parameters have been monitored in order to evaluate the four pudding formulations. The ash and moisture contents, as well as the nutritional data of the puddings as presented in Table 2 and Table 3, respectively. Differences in the nutritional data are present between formulations.

The formulations prepared with fresh fruit have similar moisture content mostly due to the cow milk and the peach purée. The other two formulations, made with lyophilized fruit have lower moisture content. The ash content in formulations CFF and RFF is practically the same, but the one in formulations CLF and RLF is significantly different. Here, not only the type of starch is responsible for this difference, but also the difference of the fruit type introduced in the pudding. The results obtained for the moisture content correspond well to those reported by other researchers [39].

### 3.2. Nutritional Data of the Nectarine-Enriched Puddings

Every change in a recipe, even the slightest, reflects the nutritional content as seen by its nutritional value. The European food systems aim at including both healthy nutrition and sustainability enabling the construction of coherent products that will be beneficial to sustainability, agriculture and human health [40]. Table 3 summarizes the nutritional data of the four formulations.

**Table 3:** Nutritional data of pudding formulations (RFF—rice starch, peach purée; RLF—rice starch, lyophilized fruit; CFF—corn starch, peach purée; CLF—corn starch, lyophilized fruit).

Pudding Formulations, 100 g	Proteins, g	Carbohydrates, g	Sugars, g	Fat, g	Monosaturated Fats, g	Energy, Kcal
RFF	2.31	10.38	3.04	2.14	1.24	70.0
RLF	3.11	13.29	4.71	3.02	1.67	93.6
CFF	2.22	10.32	3.04	2.13	1.23	69.0
CLF	3.03	13.24	4.71	3.01	1.66	93.0

Concerning the protein content of the nectarine-enriched puddings, it ranged from 2.22 (CFF) to 3.11 (RLF). The pudding formulations cannot be considered protein dense foods as they can account for only a small quantity of the needed daily protein according to factors like physical activity, age, gender, physiological needs, etc. mainly depending on the amount of milk used in the formulation. The formulations prepared with lyophilized fruit though, are compatible with inulin-enriched puddings prepared with skimmed milk [41].

Referring to the carbohydrates, the formulations prepared with lyophilized fruit had 1.3-times more carbohydrate than those with nectarine purée. Carbohydrates in the formulations are mostly presented by starch polysaccharides, the nectarine’s glucose, fructose and sucrose, and the milk’s lactose, which reflects the overall flavor of the formulations. Commercially available puddings usually contain around 20 g/100 g of carbohydrates which makes the current formulations quite appealing in terms of carbohydrate intake.

Regarding the lipid content of the formulations, it varied from 2.13 to 3.02 g/100 g. This lipid content is lower than the one in commercially available puddings bearing in mind monosaturated fats and total fat.

The consumption of excessive refined sugar contributes to total energy intake and is usually associate with events like overweight and obesity [42]. The amount of energy obtained by consuming 100 g of a pudding formulation hints that these formulations have considerably low amounts of energy 69–93.6 kcal/100 g. Following the information reported in regulation (EC)1924/2006 [43] of the European Parliament and of the Council of 20 December 200 no health claims can be made with reference to the nectarine-enriched puddings.

### 3.3. CIELAB Color Spectra of the Nectarine-Enriched Puddings

Color will always be one of the first indicators leading to the consumer’s acceptance of food products. Table 4 is a visual expression of the measured color parameters of the studied formulations, as color itself is a very important determinant.

The highest values for brightness can be found in the RFF formulation, whereas “b” (≥19.48) color parameters hint for a yellow shade. The L values of formulations CFF and RFF are highly influenced by the peach flesh color (65.13 ± 6.33) [44]. The “h” value ranging from 74.54 ± 0.89 (RLF) to 81.50 ± 1.44 (RFF) also indicates a predominating yellow color of the formulations. Its lower values, like in formulations CLF suggest the presence of an orangey shade. The natural colorants are frequently perceived by lower “c” values and higher L values [45]. This is justified by the current results indicating that the formulations have less vivid colors, but these will most likely be interpreted as more natural by consumers. For example, inulin-enriched dairy desserts studied by González-Tomás et al. [41] show higher CIELAB values for all studied parameters. Other research teams focusing on starch-based dairy desserts also report higher values for the brightness parameter [46]. This confirms that the current results differ from the typical pudding color characteristic scheme.

### 3.4. Water Activity, Water Holding Calacity and Density of the Nectarine-Enriched Puddings

The water activity is an important indicator for forecasting and controlling the shelf life of food products. The water activity, water holding capacity, and density parameters of the studied formulations are presented in Table 5.

High water activity affects the amino acid loss ratio [47]. What is more, the water activity in the puddings affects the Maillard reaction, which contributes to the darkening and the browning of the products by non-enzymatic reactions [48]. Therefore, high values of a_w_ like the formulations prepared with lyophilized fruits (0.956—0.958) are an indicator of a darker color in the orange-brown range. This is in line with the abovementioned CIELAB color of the puddings. On the other hand, high water activity affects the likelihood of microbial growth in the products. The a_w_ values are comparable to the ones published by Bchir et al. concerning yoghurt fortified with fresh and dried Spirulina [49].

Density is a good indicator of structural changes [50]. The density values of the studied formulations ranged from 0.77 to 1.17 g/cm^3^. The particle size is known to affect the density of samples showing that smaller particles have higher density value [51]. Rice starch has smaller granules compared to maize starch [52]. This proves the point that rice starch would form a denser structure compared to corn starch. The formulations with lyophilized fruit are 1.5-times denser that those with fruit purée. This also suggests that the lyophilized powder has smaller particles compared to the blended fruit.

All four formulations presented very limited WHC which suggests that they cannot hold the non-chemically bound water in the food matrix, and thus they are unstable in response to gravity. Some authors report that an alteration of the original pudding recipe may result in hysteresis loops of aqueous starch pastes [41]. Larger water holding capacity corresponded to the formulations prepared with nectarine purée. All of the samples presented a visible aqueous layer at the bottom of the glass tubes after centrifugation. These results indicate that a natural stabilizing agent is needed in each formulation to better their WHC. The substitution of cow milk with soy milk for instance, which has a better protein fraction, can possibly result in a more stable structure. Several studies suggest that soy milk is a good source of stable systems in terms of WHC and syneresis [53]. The poor WHC can also be due to the total substitution of sugar in the formulations, or to the fact that native starches have lower levels of water absorption [54]. WHC is directly linked to the number of hydrogen bonds starch forms after mixing with water [55]. Having a low capacity to hold the water may possibly be due to the fact that we do not have concentrated temperature damage on the starch granules which does not expose more available hydroxyl groups for bonding. Peak swelling of the starch granules usually occurs between 70 and 90 °C [56]. Some of the hydroxyl groups could have also bonded with the fruit itself, especially the lyophilized one, resulting in a smaller WHC.

### 3.5. Freeze-Thaw Stability of the Nectarine-Enriched Puddings

**Figure 3:**
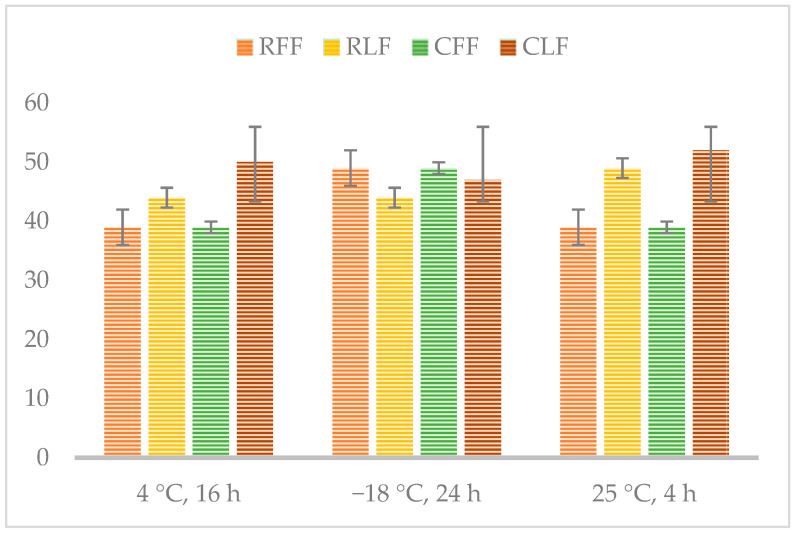
Freeze-thaw stability of pudding formulations (RFF—rice starch, peach purée; RLF—rice starch, lyophilized fruit; CFF—corn starch, peach purée; CLF—corn starch, lyophilized fruit).

Syneresis was not observed in the freshly prepared formulations, but the freeze-thaw stability of the formulations can be considered poor (Figure 3) which means that a considerable amount of water is lost during the thawing process. This undoubtedly suggests that large ice formations in the formulations during freezing affected the microstructure of nectarine-enriched puddings. In line with the limited WHC, the formulations exhibit low freeze thaw stability, proving that sizeable amounts of free water in the formulations exist, as the bound water cannot form ice crystals. Reduced syneresis is proportionally linked to an increased WHC [57]. Retrogradation is also dependent of the botanical source of the starch, temperature and molecular composition [58]. These results indicate that an increase of the temperature the nectarine-enriched puddings are prepared, can result in a better stability. The inclusion of a suitable hydrocolloid can also improve the freeze thaw stability of the current formulations, as hydrocolloids improve the physicochemistry of starch gels [59]. Other authors suggest that the syneresis can be retarded with the present of sucrose, or the appropriate type of milk [60].

### 3.6. Texture Profile Analysis of the Nectarine-Enriched Puddings

Texture analysis is used as a method for food quality control [61]. Table 6 is a visual presentation of the formulations’ texture profile analysis in terms of their firmness, cohesiveness, gumminess, springiness and chewiness.

Firmness is related to the strength of the product’s structure during compression and is the greatest force during the first compression bite [62]. According to the data in Table 6, significant changes in the texture parameters of the formulations exist. The greatest firmness is observed in formulation RLF (1.35 N), while the lowest in RFF (0.85 N). The formulations prepared with lyophilized nectarines most probably show higher firmness value because of their lower moisture content compared to the nectarine purée. The higher available moisture accounts for a reduced ability of the amylose net to harden the starch gel. Compared to milk-based desserts fortified with oat gum and k-carrageenan the currently established results for the hardness parameter practically show 2-times greater values or stronger texture [63]. The cohesiveness refers to the strength of the internal bonds of the food matrix, and the extent of the force needed to deform them before rupture [64]. As the pudding formulations have a very tender texture, the cohesiveness values vary from 0.40 (RFF) to 0.62 (CLF). The cohesiveness shows the product’s ability to bind [65]. Not only the type of fruit (lyophilized or puréed) but also the starch’s nature affect the cohesiveness of the formulations. Corn starch is associated with a better cohesiveness compared to rice starch, and the lyophilized samples help increase the cohesiveness values. This probably explains the higher gumminess which is an important characteristic of semisolid foods with a low degree of hardness and high degree of cohesiveness. Higher gumminess shows greater hardness of the sample. The nectarine-enriched formulations had gumminess values that correspond very well to the established hardness. Springiness is related to the elasticity of the sample. If springiness is high, it requires more mouth chewing energy [66]. The puddings formulations show that those prepared with lyophilized fruit are forming a more elastic structure compared to the ones prepared with nectarine purée. The nectarine-enriched puddings result in a 1.5 higher springiness values compared to other fortified milk-based desserts [63]. Chewiness is a difficult parameter to be precisely measured, as each person compresses, salivates, and grinds food pieces differently. The chewiness values of four samples varied from 0.74 to 2.91 J. Chewiness shows the relationship between the gumminess and springiness. The noticeable difference in the RFF sample is directly linked to its least values measured for gumminess and springiness.

### 3.7. Sensory Evaluation of the Nectarine-Enriched Puddings

A sensory evaluation aided in the characterization of the nectarine-enriched puddings. The panelists had to classify the samples in terms of their appearance, aroma, texture, and flavor (Table 7).

Before being able to taste the samples, the panelists had to evaluate their color, as well as their aroma. The formulations prepared with lyophilized fruit had a more orange color compared to those prepared with fruit purée where the predominant color was brown. The colors were rich enough, although they were not considered vivid. The coloring was thought to be natural in all formulations. The prevalent aroma in all formulations was the fruity one and the panelists suggested that the fruit being used was peach. In the formulations, the Bulgarian nectarine variety “Gergana” (peach with no fuzz) was used. Because of the fruits naturally sweet taste, they often come in handy in the substitution in different recipes, making fruit culinary application particularly trendy. The sweetness values can greatly differ depending on the type and concentrations of the sweetener substitute being used [67].

All formulations were considered appealing and worth trying. Taste is particularly important for the final evaluation of the food choice [68]. The taste in all formulations was fruity, and the sweet taste had a value between 7.1 and 8.0. This makes the formulations especially relevant to a child’s diet because sweetness is not enhanced in children’s nutrition. Taste is particularly vulnerable with ageing, with the sweet taste being questionably affected [69]. Formulations with reduced sugar intake might become part of the menu of those diagnosed with different types of diabetes.

In terms of consistency, the formulations prepared with lyophilized fruit had a distinct grainy feeling on the mouth compared to those prepared with fruit purée. All formulations can be considered with a semi-liquid, creamy structure. The texture was smooth with no lumps. This consistency was associated as appropriate for a child between the age of 10–16 months, and labelled as flowy, mildly thick, needing a thickener if targeting adult nutrition. Semi-liquid consistency in foods can be successfully applied in the nutrition of individuals suffering from dysphagia [70]. Tender and easy to chew food preparations can also be an accessory to the nutrition of the elderly, where different conditions may require a specific texture of the food.

### 3.8. Pearson’s Correlation Coefficients

Table 8 shows the Pearson’s correlation values between the moisture, density, water activity and texture parameters of the studied formulations. All variables showed a positive relationship, and the samples were considered to have a correlation if the correlation coefficients were ≥0.75.

Statistically independent variables, with a correlation coefficient equal to zero, are the moisture content and the springiness in the formulation with rice starch and lyophilized fruit, as well as the a_w_ and firmness, and gumminess in the formulation prepared with corn starch and fruit purée. The formulation consisting of lyophilized fruit and corn starch showed statistical independence between the gumminess and the a_w_. Variables connected by a perfectly linear relationship, with a coefficient equal to 1.000 were the moisture and springiness, and gumminess (RFF); the a_w_ and the chewiness in RFF and CFF.

The water activity had a positive correlation with the gumminess in RFF and RLF; the cohesiveness and firmness in RLF and CLF; the springiness in RFF, CFF, and CLF; the chewiness in RLF and CLF. The moisture had a positive correlation with the firmness, cohesiveness and chewiness in RFF; springiness and chewiness in CFF; firmness in CLF. The density showed positive correlations only in the formulations prepared with corn starch.

The findings of this study have to be seen in light of some limitations. As with a number of studies, the design of the current is subject to new formulations that can hardly be subjected to a reference sample. The total substitution of an ingredient and the absence of another, in this case peach incorporation in the absence of refined sugar, can be seen as initial results that can be used as reference samples for future research, aiming at refining the technology and recipe itself in order to present a better product. The current results are though referenced to similar research focusing on dairy desserts with various functional ingredients. Each of the studied parameters has been addressed to available literature in the abovementioned sections.

## 4. Conclusions

The effect of total sugar substitution in pudding recipes has been evaluated in order to meet the recent necessity of reduced sugar intake and healthier nutrition in general. Two types of formulations with lyophilized fruit and fruit purée have been prepared. Both methods of preparation resulted in successful products in line with the trending healthier desserts. A limitation of the study is identified in the results being referenced to literature and not a reference sample due to the expected extreme differences in color, overall appearance, and studied parameters. All four formulations had distinct peachy aroma. The formulations prepared with nectarine purée resulted in a better sensory perception about their texture, and better water holding capacity. All formulations can be considered with a semi-liquid, creamy structure. The texture was smooth with no lumps. At this point, the formulation prepared with lyophilized fruit and rice starch has the most promising results.

The outcomes of the current study can be used in the preparation of novel desserts with added nutritional value. Further exploration of the perspective of fruit-enriched puddings should be made in order to improve their technological and health-promoting properties.

## Figures and Tables

**Table 1:** Pudding formulations (RFF—rice starch, peach purée; RLF—rice starch, lyophilized fruit; CFF—corn starch, peach purée; CLF—corn starch, lyophilized fruit).

Type of Pudding	Whole Cow Milk, %	Rice Starch, %	Corn Starch, %	Peach Purée, %	Lyophilized Peach Powder, %
RFF	63	6	-	31	-
RLF	85	5	-	-	10
CFF	63	-	6	31	-
CLF	85	-	5	-	10

**Table 2:** Ash (%) and moisture (%) content of puddings formulations (RFF—rice starch, peach purée; RLF—rice starch, lyophilized fruit; CFF—corn starch, peach purée; CLF—corn starch, lyophilized fruit).

Puddings Formulations	Ash Content, %	Moisture Content, %
RFF	0.75 ± 0.01 ^b^	83.21 ± 0.06 ^c^
RLF	0.93 ± 0.01 ^a^	75.76 ± 0.06 ^a^
CFF	0.74 ± 0.03 ^b^	80.65 ± 0.06 ^a,b^
CLF	0.56 ± 0.01 ^c^	77.25 ± 0.06 ^b^

Different letters in the same column indicate statistically significant differences (*p* < 0.05), according to ANOVA (one-way) and the Tukey test.

**Table 4:** CIELAB color spectra of pudding formulations (RFF—rice starch, peach purée; RLF—rice starch, lyophilized fruit; CFF—corn starch, peach purée; CLF—corn starch, lyophilized fruit).

Pudding Formulations	L	a	b	c	h
RFF	69.20 ± 4.86 ^a^	2.88 ± 0.30 ^b^	19.48 ± 1.52 ^c^	19.70 ± 1.45 ^b^	81.50 ± 1.44 ^a^
RLF	58.11 ± 1.39 ^b^	6.72 ± 0.22 ^b^	24.32 ± 0.72 ^a^	25.24 ± 0.64 ^a^	74.54 ± 0.89 ^b,c^
CFF	60.45 ± 7.14 ^b^	4.23 ± 0.75 ^c^	20.65 ± 2.51 ^b^	21.10 ± 2.29 ^b^	78.16 ± 3.59 ^a,b^
CLF	56.24 ± 1.63 ^b^	7.62 ± 0.11 ^a^	26.54 ± 2.12 ^a^	27.62 ± 2.06 ^a^	73.93 ± 1.06 ^c^

Different letters in the same column indicate statistically significant differences (*p* < 0.05), according to ANOVA (one-way) and the Tukey test.

**Table 5:** Water activity (a_w_), water holding capacity (WHC) and density of pudding formulations (RFF—rice starch, peach purée; RLF—rice starch, lyophilized fruit; CFF—corn starch, peach purée; CLF—corn starch, lyophilized fruit).

Pudding Formulations	a_w_	Density, g/cm^3^	WHC, %
RFF	0.947 ± 0.001 ^a^	0.77 ± 0.06 ^b^	15.56 ± 2.25
RLF	0.956 ± 0.001 ^c^	1.17 ± 0.06 ^d^	0.49 ± 0.19
CFF	0.953 ± 0.001 ^b^	0.67 ± 0.06 ^a^	22.54 ± 3.15
CLF	0.958 ± 0.001 ^b,c^	1.10 ± 0.10 ^c^	3.66 ± 0.53

Different letters in the same column indicate statistically significant differences (*p* < 0.05), according to ANOVA (one-way) and the Tukey test.

**Table 6:** Texture profile analysis of pudding formulations (RFF—rice starch, peach purée; RLF—rice starch, lyophilized fruit; CFF—corn starch, peach purée; CLF—corn starch, lyophilized fruit).

Pudding Formulations	Firmness, N	Cohesiveness	Gumminess, N	Springiness, mm	Chewiness, J
RFF	0.85 ± 0.01 ^a^	0.4 ± 0.01 ^a^	0.34 ± 0.02 ^a^	2.17 ± 0.06 ^a^	0.74 ± 0.01 ^a^
RLF	1.35 ± 0.04 ^d^	0.51 ± 0.01 ^b^	0.84 ± 0.02 ^d^	3.47 ± 0.01 ^c,d^	2.91 ± 0.06 ^d^
CFF	0.94 ± 0.03 ^b^	0.57 ± 0.02 ^b^	0.48 ± 0.02 ^b^	2.59 ± 0.03 ^b^	1.24 ± 0.01 ^b^
CLF	1.12 ± 0.06 ^c^	0.62 ± 0.05 ^c^	0.64 ± 0.03 ^c^	3.18 ± 0.04 ^c^	2.04 ± 0.05 ^c^

Different letters in the same column indicate statistically significant differences (*p* < 0.05), according to ANOVA (one-way) and the Tukey test.

**Table 7:** Sensory attributes of pudding formulations (RFF—rice starch, peach purée; RLF—rice starch, lyophilized fruit; CFF—corn starch, peach purée; CLF—corn starch, lyophilized fruit).

Pudding Formulations	Color		Aroma			Consistency				Taste		
	Orange	Brown	Milky	Starchy	Fruity	Flowy	Creamy	Grainy	Thick	Sweet	Milky	Fruity
RFF	4.5 ± 1.27 ^c^	5.5 ± 1.35 ^b^	3.1 ± 1.19 ^a^	2.2 ± 1.03 ^a^	6.7 ± 0.94 ^b^	6.2 ± 1.39 ^a^	7.3 ± 0.94 ^b^	2.2 ± 0.63 ^c^	4.5 ± 1.08 ^a^	7.1 ± 0.99 ^a^	4.3 ± 1.88 ^a^	8.5 ± 1.08 ^a^
RLF	8.6 ± 1.07 ^a^	8.8 ± 0.92 ^a^	2.7 ± 1.15 ^c^	2.7 ± 1.16 ^a^	9.0 ± 1.15 ^a^	6.1 ± 1.19 ^a^	8.8 ± 1.03 ^a^	8.2 ± 0.78 ^a^	3.4 ± 1.42 ^a^	7.5 ± 1.08 ^a^	4.5 ± 1.58 ^a^	9.6 ± 1.17 ^a^
CFF	6.9 ± 0.99 ^b^	8.1 ± 1.37 ^a^	2.8 ± 1.03 ^c^	2.6 ± 1.17 ^a^	7.5 ± 0.97 ^b^	6.9 ± 1.19 ^a^	9.7 ± 0.95 ^a^	3.8 ± 2.15 ^b^	3.3 ± 1.63 ^a^	8.0 ± 1.25 ^a^	4.1 ± 1.85 ^a^	8.8 ± 0.92 ^a^
CLF	8.0 ± 1.05 ^a,b^	7.4 ± 1.07 ^a^	4.5 ± 0.97 ^b^	2.5 ± 1.26 ^a^	9.5 ± 0.85 ^a^	6.6 ± 1.51 ^a^	9.8 ± 0.78 ^a^	8.5 ± 1.08 ^a^	3.4 ± 1.65 ^a^	7.7 ± 1.25 ^a^	4.3 ± 1.71 ^a^	8.2 ± 1.68 ^a^

Different letters in the same column indicate statistically significant differences (*p* < 0.05), according to ANOVA (one-way) and the Tukey test.

**Table 8:** Pearson’s correlation coefficients of moisture, a_w_, density and texture parameters of pudding formulations (RFF—rice starch, peach purée; RLF—rice starch, lyophilized fruit; CFF—corn starch, peach purée; CLF—corn starch, lyophilized fruit).

Parameters	Firmness	Cohesiveness	Gumminess	Springiness	Chewiness
	**RFF**				
**moisture**	0.750	0.750	1.000	1.000	0.750
**a_w_**	0.250	1.000	0.750	0.750	1.000
**density**	0.328	0.179	0.007	0.007	0.179
	**RLF**				
**moisture**	0.324	0.250	0.250	0.000	0.250
**a_w_**	0.818	0.750	0.750	0.250	0.750
**density**	0.007	0.000	0.000	0.250	0.000
	**CFF**				
**moisture**	0.206	0.003	0.206	0.997	0.794
**a_w_**	0.000	0.250	0.000	0.750	1.000
**density**	0.637	0.985	0.637	0.015	0.363
	**CLF**				
**moisture**	0.997	0.206	0.297	0.206	0.206
**a_w_**	0.750	0.750	0.000	0.750	0.750
**density**	0.250	1.000	0.250	1.000	1.000

## Data Availability

The data presented in this study are available on request from the corresponding author.
